# Improving access to high-quality primary care for socioeconomically disadvantaged older people in rural areas: a mixed method study protocol

**DOI:** 10.1136/bmjopen-2015-009104

**Published:** 2015-09-16

**Authors:** John A Ford, Andrew P Jones, Geoff Wong, Allan B Clark, Tom Porter, Tom Shakespeare, Ann Marie Swart, Nicholas Steel

**Affiliations:** 1 Department of Public Health and Primary Care, Norwich Medical School, University of East Anglia, Norwich, UK; 2 Nuffield Department of Primary Care Health Sciences, University of Oxford, London, UK; 3 Norwich Clinical Trials Unit, Norwich Medical School, University of East Anglia, Norwich, UK

**Keywords:** HEALTH SERVICES ADMINISTRATION & MANAGEMENT, PRIMARY CARE, PUBLIC HEALTH

## Abstract

**Introduction:**

The UK has an ageing population, especially in rural areas, where deprivation is high among older people. Previous research has identified this group as at high risk of poor access to healthcare. The aim of this study is to generate a theory of how socioeconomically disadvantaged older people from rural areas access primary care, to develop an intervention based on this theory and test it in a feasibility trial.

**Methods and analysis:**

On the basis of the MRC Framework for Developing and Evaluating Complex Interventions, three methods will be used to generate the theory. First, a realist review will elucidate the patient pathway based on existing literature. Second, an analysis of the English Longitudinal Study of Ageing will be completed using structural equation modelling. Third, 15 semistructured interviews will be undertaken with patients and four focus groups with health professionals. A triangulation protocol will be used to allow each of these methods to inform and be informed by each other, and to integrate data into one overall realist theory. Based on this theory, an intervention will be developed in discussion with stakeholders to ensure that the intervention is feasible and practical. The intervention will be tested within a feasibility trial, the design of which will depend on the intervention. Lessons from the feasibility trial will be used to refine the intervention and gather the information needed for a definitive trial.

**Ethics and dissemination:**

Ethics approval from the regional ethics committee has been granted for the focus groups with health professionals and interviews with patients. Ethics approval will be sought for the feasibility trial after the intervention has been designed. Findings will be disseminated to the key stakeholders involved in intervention development, to researchers, clinicians and health planners through peer-reviewed journal articles and conference publications, and locally through a dissemination event.

**Strengths and limitations of this study**

- Use of a mixed methods design involving a realist review, analysis of the English Longitudinal Study of Ageing, and qualitative focus groups and semistructured interviews to develop theory.
- Triangulation and integration of data using a realist perspective.
- Key stakeholders contributing to intervention development.
- Testing of developed intervention within a feasibility trial.
- Concepts identified in the realist review may not be testable in the secondary analysis of the English Longitudinal Study of Ageing because of the variables available.

## Introduction

The UK, like most high-income countries, has an ageing population, with the number of over 65-year-olds set to increase by 9 million over the next 35 years.^1^ An ageing population presents a number of challenges, such as an increasing number of people with chronic health problems and the inevitable impact on healthcare resources. The largest study from the USA, which included over 30 million patients, estimated the prevalence of multimorbidity (two or more chronic diseases) to be 62% in the age group 65–74 years, 76% in the age group 75–84 years, and 81% for those aged over 85 years.^2^

In particular, rural areas have seen an increase in older people, with those aged over 85 years being the fastest growing population.^3^ Deprivation is high in this population with a sixth of rural pensioners living below the poverty threshold (below 60% of median income).^3^ However, it should be noted that some rural communities are wealthy with pockets of deprivation, especially along the so-called ‘urban tracks’ which provide easy access to major cities.^4^

### Access to healthcare

A review of equality of access to healthcare in the UK found that rural individuals, older people and socioeconomically disadvantaged groups have reduced access to care.^5^ Possible explanations for this may include geographic isolation, with one in five older people in rural areas living more than 4 km from their primary care provider^6^; poor transport availability, with one in three pensioner households not having access to a car^6^; and the association with deprivation.^7^ The triple jeopardy of age, rurality and deprivation leads to increased morbidity but decreased access; an example of the well-known ‘inverse care law’ that states healthcare provision is inversely related to need.^8^

Access is a complex concept leading to several previous studies using service utilisation as a proxy end point.^9–11^ This is conceptually different from access because an individual could have good access but may never need to use a service. Researchers have tried to theorise access and most of the theoretical research has been dominated by the Andersen model of predisposing (eg, age, sex and social structure), enabling (eg, distance to healthcare) and need (eg, symptoms and functioning) factors.^12^ Ricketts and Goldsmith reviewed the different concepts which have been used to define access in the literature and conceptualised it as dynamic, thus acknowledging the balance between health service need (patient side) and health service use (provider side).^13^ They argue that the concept of access is not linear but an iterative process of both patients’ learning from prior attempts and their changing perception of need.

Despite its complexity, improving access to primary care has been increasingly topical with some believing that it will reduce secondary care use.^14^ Soljak *et al* found some evidence for this in an English national cross-sectional study with over 52 million participants. They found that improved access to primary care reduced stroke admissions.^15^ Furthermore Cowling *et al*^16^ undertook a study of 7856 patients in England and found that good patient-reported access to primary care was associated with lower self-referred emergency department attendances.

With a view to improve access to healthcare, a recent major systematic review listed barriers to accessing primary care.^17^ These were categorised as patient factors (eg, sociodemographic), organisational factors (eg, appointment system), financial factors, workforce factors (eg, technical skills) and geographical factors. However, the review failed to consider the dynamic and iterative concept of access that balances provider-side and patient-side components.^13^

Interventions to overcome barriers to access have been assessed in two recent major systematic reviews. Interventions employed included walk-in centres, reminder systems, text messaging, multilingual services, telephone consulting, and advanced access initiatives.^17^ ^18^ It was found that interventions with multiple linked strategies targeted at different levels of the healthcare system were more likely to be effective. The authors found most interventions were not targeted and there was a lack of research on specific population groups.^17^ Initiatives that increase access to primary care for the whole population, such as walk-in centres with extended opening hours, have been criticised because they have the potential to increase access for the worried well and create demand without improving outcomes or efficiency.^19^ ^20^

### Mixed methods research

Both quantitative and qualitative approaches are required in order to obtain a full understanding of the problems underlying access to care; yet it is noteworthy that most previous research has taken a quantitative approach. Few studies have employed qualitative methods,^21–23^ with even fewer using mixed methods.^24^ Mixed method research is challenging, including the difficulty of reconciling differing philosophical paradigms, commonly referred to as ‘paradigm wars’.^25^ Quantitative approaches usually assume a positivist perspective and qualitative approaches take a interpretivist or constructivist perspective; Giddings and Grant criticise the manner in which mixed method research combines philosophical paradigms, suggesting it is a ‘Trojan Horse for positivism’.^26^ Johnson *et al*^27^ suggested that pragmatism offers the most appropriate philosophical approach for mixed methods, but pragmatism itself is subject to substantial philosophical ambiguity. Realism, of which critical realism is a part, is ‘the view that entities exist independently of being perceived, or independently of our theories about them’.^28^ A realist approach is particularly useful in mixed methods because it complements the synthesis process by allowing different techniques to confirm or refute findings. It provides a consistent philosophical paradigm for mixed methods research that allows both quantitative and qualitative data to be used under one paradigm in the service of developing a realist programme theory of middle-range abstraction. A key underlying principle of realism is that researchers cannot have certain knowledge of the world or objectivity, but that all knowledge is partial and fallible, and therefore theory generated from a realist perspective is only as good as it is until it is disproved.

A further criticism of mixed methods research has been the lack of successful integration of data.^29^ Proposed solutions include triangulation protocols, following a thread and mixed method matrix.^30–32^ Each of these methods integrates data at different stages; triangulation protocols integrate data at the interpretation stage, whereas following a thread and mixed method matrix combines data at the analysis stage. While triangulation protocols have been used in previous research, detailed descriptions of the process used are rare.^30^

Dowrick *et al*^24^ developed an intervention to improve access to primary care mental health using mixed methods. The authors first synthesised evidence from scoping reviews, secondary analysis of qualitative data, stakeholder dialogues, and services user and carer interviews to understand the problems and develop solutions. Based on their findings, the authors developed a three-component model to improve access which included community engagement, primary care quality, and tailored psychosocial interventions. The subsequent evaluation found that a multilevel intervention incorporating these three components was most effective.

### Aims and justification

The aim of this study is to develop theory around how socioeconomically disadvantaged older people from rural areas access primary care, develop an intervention, and then to test it in a feasibility trial. We presented a protocol, building on the methodology used by Dowrick *et al*,^24^ for a mixed method study which synthesises evidence across qualitative and quantitative methods using a realist perspective, integrates data by using a triangulation protocol and develops an intervention to be tested.

## Methods and analysis

**Figure 1:**
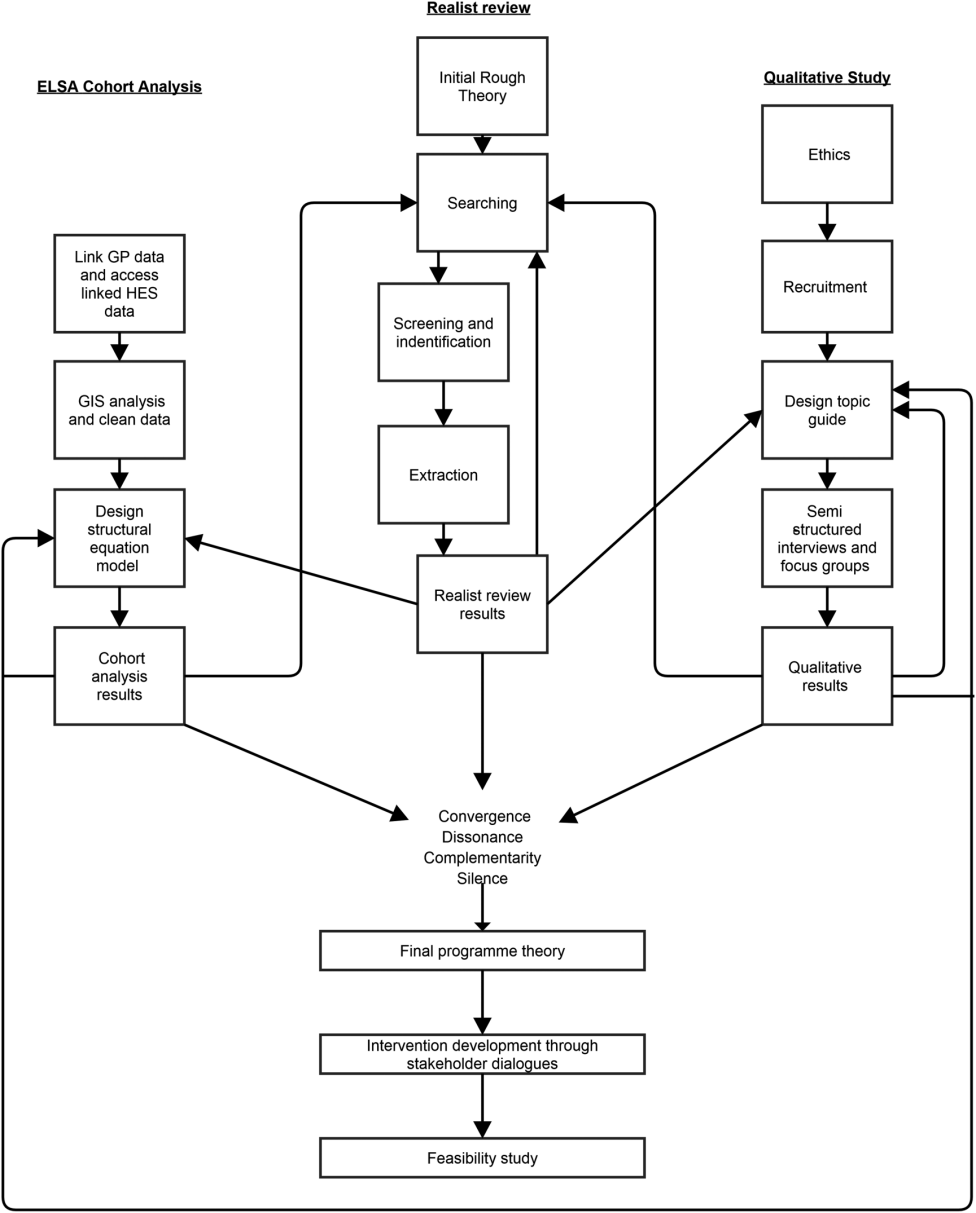
Triangulation protocol. (ELSA, English Longitudinal Study of Ageing; GIS, Geographic Information System software; GP, general practitioner; HES, Hospital Episode Statistics).

The MRC Framework for Developing and Evaluating Complex Interventions will be used to guide the research.^33^ First, theory will be generated using three contrasting but complementary methods to explore the barriers and facilitators to accessing high-quality primary care for socioeconomically disadvantaged older people in rural areas. The three methods used will be realist literature review, secondary analysis of the English Longitudinal Study of Ageing (ELSA), and qualitative focus groups and interviews. Robust integration of these data will be paramount and figure 1 shows a triangulation protocol detailing how these data will be integrated. A realist approach will be taken to synthesise and integrate data.^34^ This theory will be explored with stakeholders to develop an intervention which will be tested and refined in a feasibility trial.

### Realist review

A realist review not only allows for the development and refinement of a ‘pathway’ (in realist reviews this is called a programme theory) but also allows for unearthing of the causal processes behind the programme theory (through an analysis of contexts, and mechanisms and outcomes).^35^ This is ideally suited to understand the complexities of the dynamic and iterative concept of primary care access as a balance of patient-side and provider-side components. Realist review focuses on answering questions such as ‘how?’, ‘why?’, ‘for whom?’, ‘in what circumstances?’ and ‘to what extent?’ access might lead (or not lead) to changes in quality of care and/or clinical outcomes. Therefore, unlike traditional systematic reviews that concentrate on making judgements (eg, which intervention is more effective?), realist reviews focus on explanations and understanding.

Initial ‘rough’ programme theory will be generated based on informal searches of the literature, and personal content expertise and understanding of the problem. A more formal literature search will be undertaken in MEDLINE, MEDLINE in process and EMBASE from inception to seek out data to refine the initial ‘rough’ programme theory. Search terms that will be used are shown in online supplementary appendix A. There will be no restriction on study design. Grey literature will be searched using an internet search engine and there will be a targeted search of specific websites. All titles and abstracts will be screened. Articles will be included if they consider how socioeconomically disadvantaged older people access care. Studies will not be restricted to rural areas since the barriers individuals face in rural areas may not be unique and therefore, may be covered in broader studies. Only studies from high-income countries will be included. Pawson's^34^ concepts of relevance and rigour will be used to guide document selection. Data from included studies will be coded in QRS NVivo—with coding being both inductive (drawn from the data in the included documents) and deductive (drawn from the programme theory). Analysis and synthesis will focus on (1) assigning conceptual categories to the codes (ie, are these data about context, mechanism or outcome); (2) use of the data to configure context, mechanism and outcome configurations (CMOCs) and (3) use of the data to understand the place and relationships of the CMOCs with the programme theory. The realist review's product will be a realist programme theory that is middle range in abstraction—that is a programme theory that has been empirically tested against data from included documents and is at a level that is testable. During the refinement of the realist programme theory, we will continue to undertake purposive focused searches informed by the programme theory to seek out relevant substantive theory to corroborate and/or add explanatory power. Where relevant, any substantive or formal theory identified from included documents (eg, locus of control^36^) will be analysed to determine if it is relevant to and can add further explanatory power to the realist programme theory we will develop. Reporting of the realist review will adhere to the RAMESES publication standards for realist reviews.^37^

### Analysis of the ELSA

Findings from the realist review will be explored within the ELSA. ELSA is a national, longitudinal, face-to-face interview study of older people aged 50 years and over, initially living in private households. Data that cover health, functioning, social participation and economic position are collected every 2 years with biological and anthropometric information gathered every 4 years. First data collection was in 2002, and the latest data collected (2012/2013) has information on approximately 17 000 individuals, of which over 5000 have participated in all possible interviews.

In 2013, ELSA was linked with the Hospital Episode Statistics (HES). HES consists of routinely collected secondary care data and contains admissions, outpatient appointments, and A&E attendances in NHS hospitals in England. This enables routinely collected clinical outcomes to be analysed alongside the wealth of participant-reported data in ELSA. Added to this linked data set will be road distance and car travel time from a participant's home to general practitioner (GP) practice, which will be calculated using Geographic Information System software.

Practice level data will be added from the GP Patient Survey and Health and Social Care Information Centre (HSCIC). The GP Patient Survey collects annual data on patient experience in all general practices in England and was initially established to monitor the NHS from the patient's perspective by collecting a range of patient access factors. Rural index values (combination of average distance from a patient's home to their GP and average population density), deprivation, practice size and Quality Outcomes Framework (QoF indicators) will be added from the HSCIC.

Structural equation modelling (SEM) will be used, based on the theory from the realist review, to explore access in the ELSA cohort which is linked with HES and GP practice data. SEM will be constructed to examine the relationship between access factors, quality of care and secondary care use. SEM allows for theories to be constructed and explored statistically.^38^ Primary analysis will be undertaken cross-sectionally using data collected from the most recently available point, wave 6 (2012), and then subsequently by using the longitudinal data set.

### Semistructured interviews and focus group

Semistructured interviews will take place with older people and focus groups with health professionals to: explore experiences of older people in accessing primary care, discuss findings from the realist review and examine the results from the ELSA analysis.

Fifteen older people who receive a means tested benefit and live in a rural area will be recruited from two communities with a high number of deprived households, pension credit claimants and rurality (based on local authority data). Invitation cards and posters will be distributed in community amenities and groups, such as post offices, grocery stores, public houses, pharmacies, churches and bowls clubs. A purposive sampling frame will be employed to ensure at least three participants are male, two participants are over 80 years of age, and four participants are from different practices to ensure that the interviews are not dominated by any one population group. Participants who are unable to give informed consent will be excluded. Semistructured interviews will last approximately 1 h.

Two focus groups will be undertaken with GPs, healthcare planners and community geriatricians, and two will be undertaken with district nurses, community matrons and case managers. Participants will be recruited through local health providers and the East of England National Institute for Health Research Clinical Research Network. There will be five to six individuals in each of these four focus groups which will last approximately for 2 h.

The topic guide for the interviews and focus groups will be designed based on the results of the realist review. It will start with open-ended questions and progress to more focused questions around findings from the realist review. Hypothetical patient vignettes will be used to explore realist themes. The interviews and focus groups will be audio recorded and transcribed. Data will be analysed using thematic analysis, using an inductive approach, in QSR NVivo.

### Triangulation protocol

The realist review, ELSA analysis and qualitative component will all explore the contexts that positively or negatively influence access to high-quality primary care for socioeconomically disadvantaged older people in rural areas, but from different perspectives. Each technique will be informed by, and contribute to, each other. The means by which each method will ‘talk to each other’ is shown in the triangulation protocol in figure 1. Use of a triangulation protocol has been recommended to integrate mixed methods data.^39^ The results from each method will then be synthesised together to form one overarching realist programme theory. By looking for convergence (same results), dissonance (opposing results), complementarity (supportive or explanatory results, but not the same) or silence (no evidence to support or refute) we will be able to further test and refine parts of the overarching programme theory.

### Intervention development

The integrated theory will contain CMOCs, as per the realist methodology.^34^ The intervention will aim to modify contexts in order to affect mechanism and subsequent outcomes. An iterative process will be used, based on the interventions from the literature and contexts which could be influenced, to design an initial intervention. As used elsewhere, this intervention will be developed further through stakeholder dialogues.^24^ This will involve discussing the results and possible interventions with key stakeholders. Key stakeholders will include NHS England, Norfolk Older People's Strategic Partnership, HealthWatch and local Clinical Commissioning Groups. A dialogue analysis template will be created for each encounter and this will be sent back to relevant stakeholder to check for accuracy. The development of the intervention will be tracked to allow a clear understanding of how and why changes have been made. This iterative technique will ensure that the intervention is practical and feasible with face validity.

### Feasibility trial

The design of the feasibility trial will depend on the intervention developed. If the intervention aims to target groups (such as primary care providers) rather than individuals, a cluster design will be used.^40^ The purpose of the feasibility trial will be to gather the information needed for a definitive trial, and optimise the implementation and use of the intervention. Parameters measured within the feasibility trial will include recruitment and retention, practicality of collecting outcome measures, completeness of data collection, and data required for the assessment of cost-effectiveness. The trial and intervention will be reported according to CONSORT and TIDieR guidelines.^41^ ^42^

## Discussion

This research aims to develop a specific intervention to improve access to primary care for socioeconomically disadvantaged older people in rural areas. Based on the MRC Framework for Developing and Evaluating Complex Interventions, it uses a mixed method approach to provide a coherent and plausible theoretical basis to inform intervention development from a realist perspective. Realist review, ELSA cohort analysis, and qualitative focus groups and interviews are used to explore the contexts that influence access to high-quality primary care for socioeconomically disadvantaged older people in rural areas. These findings will be discussed with stakeholders in order to design an intervention. Finally the intervention will be tested within a feasibility trial.

### Strengths and limitations

This study uses three methods to look at the same research question, providing corroboration and exploration of findings leading to comprehensive understanding of the issue. This corroboration is consistent with the one philosophical paradigm that is used throughout the mixed methods, realism. Realism highlights the need for theory to be falsified or supported by evidence.^34^ The three methods used in this research will allow for theory to be checked for convergence or dissonance. Using a clear and transparent triangulation protocol not only allows for this integration but also enables communication during data collection.

ELSA is a large cohort study established to measure a range of social determinants of health alongside health outcomes in older people, providing a rich source of data to explore barriers to healthcare. Linked with this data set will be hospital data at an individual level and primary care data at a practice level as contextual variables leading to a wealth of data on the patient care pathway.

SEM will be used to analyse theory generated from the realist review. The ability to statistically model theory generated in this way will allow corroboration of results; however, not all concepts identified in the realist review may be able to be tested in the linked ELSA data set. Latent variables may need to be created or concepts excluded to address this problem.

This data will be used in discussions with stakeholders to ensure that the intervention developed is practical, feasible and acceptable. Lessons from the feasibility trial will be used to refine the intervention and gather the information needed for a definitive trial such as practicability of the intervention, recruitment and retention rates and effect sizes, and variance required for a sample size calculation.

### Potential impact

Improving access to primary care for socioeconomically disadvantaged older people in rural areas will hopefully help these individuals better utilise their primary care provider. We anticipate that this will have a positive impact on adherence to chronic disease management and will likely help them access the correct urgent care service at an early stage when they become unwell. Preventative measures may then be potentially started earlier, reducing hospital admissions and pressure on urgent care services. In turn, this should then reduce health inequalities.

## Ethics and dissemination

Ethics approval from the regional ethics committee has been granted for the focus groups with health professionals and interviews with patients. Ethics approval will be sought for the feasibility trial after the intervention has been designed.

Key stakeholders will be made aware of the research through the stakeholder dialogues. The findings of the research will be reported back to each of them. Results will be disseminated to researchers, clinicians and health planners in peer-reviewed journal articles and conference publications. One or more dissemination events will be held locally to feedback to participants and contributors to the research.
